# Factors Influencing High School Students’ Interest in pSTEM

**DOI:** 10.3389/fpsyg.2018.01535

**Published:** 2018-08-21

**Authors:** Tiffany A. Ito, Erin McPherson

**Affiliations:** Department of Psychology and Neuroscience, University of Colorado Boulder, Boulder, CO, United States

**Keywords:** STEM education, gender, gender disparities, belonging, self-efficacy

## Abstract

The transition from high school to college is an important choice point for the pursuit of physical science, technology, engineering, and mathematics (pSTEM) career paths, with students in the United States switching from course selection that is proscribed by state graduation requirements in high school to choosing classes and paths of study more freely in college. Here two studies examine whether social factors identified to inhibit interest in pSTEM within college students similarly affect high school students, and in particular whether these factors could contribute to gender differences in interest in pursuing pSTEM. We find a lower sense of social and ability belonging and lower self-efficacy among female than male high school students pursuing pSTEM classes. In addition, for females but not males, social belonging significantly predicts intentions to continue to pursue pSTEM, highlighting the importance of considering whether low social belonging inhibits intentions to pursue pSTEM for female but not male students. We also find that perceptions of pSTEM fields as requiring innate brilliance more than hard work selectively discourage female students from intending to further pursue pSTEM. Together the studies highlight the potential impact of both subjective self-perceptions and perceptions about pSTEM fields on students’ interest in pSTEM and further identify processes that may selectively dissuade high school females from pursuing pSTEM career paths relative to males.

## Introduction

This paper addresses the broad issue of women’s underrepresentation in physical science, technology, engineering, and mathematics (pSTEM) by examining factors that affect intentions to pursue pSTEM among high school females and males. Our interest is motivated by the historical underrepresentation of women in these domains, particularly in North America; whereas roughly equal numbers of women and men pursue college degrees and employment in the biological sciences – and women tend to outnumber men in behavioral and health science fields – the participation of women is still far lower than that of men within the specific specialties of physical sciences as well as math, engineering, and computer science. This can be seen within higher education, where women receive only a small fraction of undergraduate degrees in physics (19%), engineering (20%), and computer science (18%) (National Science Foundation [NSF], 2015). Disparities continue in the workforce, with women constituting 12% of physicists/astronomers, 15% of engineers, and 24% of computer and information scientists (National Science Foundation [NSF], 2015).

Given the striking gender disparities in pSTEM within higher education and beyond, it is not surprising that many studies focus on the factors that predict pSTEM pursuit and persistence at the college level. However, it is also important to study these processes earlier in the educational pipeline, including among high school students on the cusp of transitioning to higher education and/or the workforce. Research shows a high drop-off in pSTEM pursuit at this structural transition; high school students have experience with pSTEM via required coursework and electives, yet relatively few choose to major in a pSTEM field in college. As an example, recent data show that 72.4% of female and 66.7% of male United States high school students take chemistry (Cunningham et al., 2015), yet very few incoming college freshman intend to major in a physical science (e.g., 2.2% in 2012) (National Science Foundation [NSF], 2014). The two studies reported here assess whether some of the social factors identified to inhibit interest in pSTEM within college students similarly affect high school students, and in particular whether these factors could contribute to gender differences in interest in pursuing pSTEM^1^.

One critical social factor implicated in both students’ performance and persistence in pSTEM is their feeling of acceptance and fit within pSTEM (Lewis et al., 2017). Belonging is a fundamental innate human need which, when met, facilitates physical and psychological well-being (Maslow and Lowry, 1968; Baumeister and Leary, 1995; Hawkley and Cacioppo, 2010), including academic achievement and persistence. Studies measuring belonging at the general school level find a positive association between belonging and higher expectations of success, school motivation, self-reported effort, and grades (Goodenow and Grady, 1993; Anderman and Freeman, 2004; Hausmann et al., 2007; Walton and Cohen, 2007; Pittman and Richmond, 2008; Zumbrunn et al., 2014). More focal measures of belonging within a specific discipline predict discipline-specific achievement. Of particular relevance for the present paper, studies measuring pSTEM belonging show that a higher sense of fit and acceptance within pSTEM is associated with higher grades in pSTEM courses, higher intentions to continue in the field, and increased likelihood of actual persistence (Murphy et al., 2007; Good et al., 2012; Smith et al., 2013; Lewis et al., 2017; Banchefsky et al., unpublished; see also Walton et al., 2012).

Although belonging facilitates academic success for both men and women, within pSTEM, women often report lower levels of belonging than men (Murphy et al., 2007; Lewis et al., 2017; Banchefsky et al., unpublished). This raises the question of whether gender differences in belonging contribute to gender disparities in these fields. Consistent with this possibility, research shows that students who are at the greatest risk of failure in academic settings and/or who have the greatest belonging concerns are the most sensitive to contextual cues signaling their status (Walton and Cohen, 2007; Hughes et al., 2015; Lewis et al., 2017). Since women are likely to have more reasons to question their status in pSTEM than men (e.g., due to their underrepresentation or negative culture stereotypes about their ability; Cheryan et al., 2017), these findings predict that perceived belonging will more strongly influence pSTEM persistence for females than males. Consistent with this prediction, multiple studies show a stronger connection between sense of belonging and pSTEM persistence for women than men at the college and graduate education level (Lewis et al., 2017; Banchefsky et al., unpublished).

In addition to students’ subjective self-perceptions of belonging, other studies show the influence of students’ perceptions about pSTEM fields on their interest in those fields. McPherson et al. (2018), for instance, found greater interest in pSTEM among college students who perceive a smaller discrepancy between self and the typical pSTEM student on dimensions like *scientific* and *meticulous*. The degree to which a field is perceived as requiring innate brilliance as opposed to dedication and hard work also influences interest, especially among women. For women, but not for men, interest is lower for careers and college majors associated with brilliance (Bian et al., 2018). Since pSTEM fields are among those assumed to require innate ability more so than hard work (Leslie et al., 2015; Meyer et al., 2015), these perceptions about the fields could selectively discourage female students.

## Study 1: Gender Differences in pSTEM Belonging

Two studies are presented that examine how self-perceptions and perceptions about pSTEM affect high school students’ interest in pursuing pSTEM. Study 1 focuses on self-perceptions, closely mirroring research done at the college level to examine (1) whether high school females feel a lower sense of belonging in their pSTEM classes than high school males, and (2) whether belonging is a stronger predictor of intentions to pursue pSTEM for high school females than males (cf. Lewis et al., 2017; Banchefsky et al., unpublished). These predictions, derived from prior research primarily with college students, rest on the assumption that high school and college students experience similar influences relevant to pSTEM belonging (Lewis et al., 2017) such as cultural stereotypes that women and girls are less adept at math and science than men and boys (Nosek et al., 2002; Robinson-Cimpian et al., 2014). Developmental research indicates that children as young as second grade more strongly associate math with boys than girls (Cvencek et al., 2011), suggesting the effects of these stereotypes will be evident in our high school sample. Another factor common to high school and college contexts is the relatively lower number of female than male peers and role models in pSTEM. Although the student gender distribution is more equal in some high school classes such as calculus and chemistry, males still typically outnumber females in other high school pSTEM classes like physics and engineering (Cunningham et al., 2015). Similarly, while the representation of women among high school science teachers has increased, they are still in the numerical minority and the gap is particularly large in some disciplines (e.g., physics; Blank and Langesen, 2003). When considered together, these various factors suggest that the social climate facing high school females subtly (and perhaps not-so-subtly) leads them to question whether pSTEM is the place for them, resulting in lower mean levels of belonging among females than males. Moreover, past research showing that students with greater concern about their belonging are the most sensitive to belonging cues (Walton and Cohen, 2007; Hughes et al., 2015; Lewis et al., 2017; Banchefsky et al., unpublished) suggests that belonging will be a stronger predictor of intentions to persist in pSTEM for high school females than males.

We assessed our two hypotheses by sampling high school students currently taking a pSTEM course. Of primary interest was their subjective sense of belonging within that pSTEM course, and their interest and intentions in pursuing pSTEM in the future. We were unable to obtain identifiable data that would allow us to track students’ actual course-taking behavior, but intentions are a proximal predictor of behavior (Ajzen, 1985, 1987, 1991, 2011) and consequently a frequently used measure of educational outcomes (e.g., Tinto, 1993; Murphy et al., 2007; Good et al., 2012; Lewis et al., 2017; Banchefsky et al., unpublished).

In addition to examining the role of belonging in pSTEM at the high school level, Study 1 also expands prior research by considering multiple forms of belonging. Prior studies have tended to leave the specific type of belonging unspecified or focus on social relations in particular (e.g., Murphy et al., 2007; Good et al., 2012; Lewis et al., 2017). The prior results linking belonging and persistence in pSTEM, especially for women, indicate these forms of belonging affect persistence. However, Lewis and Hodges (2015) propose that overall academic belonging involves both a more social component and an ability component, and find that both social and ability belonging independently predict intentions to persist academically. Similarly, others have shown that perceptions of needing to exert more effort than peers to succeed are higher for women than men, and these perceptions decrease academic motivation for women but not men (Smith et al., 2013; Banchefsky et al., unpublished). Together, these findings suggest the importance of explicitly considering not only perceptions of social fit, but also fit along the academic/ability dimension. Work on ability fit has so far focused on college students; here we ask whether high school females in pSTEM classes are similarly more concerned about how their abilities compare to their peers than their male counterparts are, resulting in lower ability belonging in pSTEM among high school females.

In addition to social and ability belonging, we also consider a general sense of school belonging to assess the relative importance of acceptance in school generally, versus within a specific academic domain. Doing so allows us to examine a general gender role explanation for prior findings that pSTEM persistence is more influenced by social belonging for females than males: that females are simply more sensitive to social cues in general. This possibility derives from women’s socialization to attend to interpersonal harmony and social relations (e.g., Cross and Madson, 1997). Women also more strongly endorse communal goals and an interdependent sense of self compared to men (Twenge, 1997; Diekman et al., 2011). Prior gender differences in pSTEM belonging and its relation to persistence might thus reflect a greater orientation to social cues among females than males in general, rather than something specific to pSTEM. If this is the case, gender differences in belonging, and the greater relation of belonging to persistence among females than males may occur across all measures of belonging (i.e., pSTEM-specific and general school belonging). By contrast, if the lower social belonging observed among women in pSTEM in past research reflects something specific about their experiences in pSTEM, we would expect gender differences favoring high school males only on the pSTEM-specific belonging measures and not general school belonging, and the stronger relation between belonging and persistence for high school females only for pSTEM-specific belonging.

Finally, another strength of Study 1 is the inclusion of self-efficacy, or beliefs about one’s ability to plan for and execute steps necessary for future success (Bandura, 1986). Efficacy promotes academic performance and motivation (Lent et al., 1986), and girls often experience lower self-efficacy than boys (Orenstein, 1994). In pSTEM in particular, women often report lower pSTEM self-efficacy than men (Pajares and Miller, 1994; Vogt et al., 2007; Kost-Smith et al., 2009; Stout et al., 2011; Lewis et al., 2017), and some have pointed to this disparity as contributing to women’s relatively worse pSTEM performance as compared to men (Pajares and Miller, 1994; Cheryan et al., 2017). Including self-efficacy provides a rigorous test of the degree to which belonging in any of its forms predicts persistence. Note that while self-efficacy and ability belonging each involve perceptions of ability, efficacy is typically assessed as absolute confidence in one’s ability to complete specific tasks. Ability belonging, by contrast, reflects assessment of one’s overall ability relative to peers. They are typically moderately related but differentially related to academic outcomes (Lewis and Hodges, 2015; Banchefsky et al., unpublished), highlighting the benefits of assessing each.

### Study 1 Materials and Methods

#### Subjects

Subjects were 127 high school students taking different pSTEM-based classes (advanced chemistry, Advanced Placement physics, robotics, Advanced Placement Java, design technology, geometry, advanced precalculus, and Advanced Placement calculus) from a single high school (53 females and 74 males). Fourteen additional students (7 females and 7 males) enrolled in biology who completed surveys were omitted from analyses. Since females and males are more equally represented in the biological sciences (e.g., females constitute 59% of graduates with a bachelor’s degree in biology; National Science Foundation [NSF], 2015), we do not expect the same gender differences in belonging within biology classes. Students were invited to participate based on their enrollment in any science class, so it was not possible to identify only those in physical and not biological science courses in advance. Self-reported race within the final sample was as follows: among females, 44 identified as White, 6 as Asian/Asian American, 2 as Latina/Hispanic, and 1 as Native American; among males, 58 identified as White, 1 as Black/African American, 8 as Asian/Asian American, 4 as Latino/Hispanic, and 2 as Native American. Because we do not have access to rosters of the classes from which subjects were recruited, we are unable to compute response rates.

Both Study 1 and 2 report analyses of existing data collected anonymously by a high school student for educational purposes as part of a requirement for a research experience class; such class projects are not considered research by the University of Colorado, Institutional Review Board (IRB). After completion of the class projects, the first author and the student who completed the project discussed disseminating the findings, at which point the IRB at the University of Colorado was consulted again. They determined that analyses of these existing anonymous data do not constitute research involving human subjects as defined by the US Department of Health and Human Services and Federal Drug Administration.

Written informed consent was not obtained since both studies were conducted originally for educational rather than research purposes. At the time surveys were completed, students were simply asked if they wanted to complete an anonymous survey for a fellow student’s research experience class. They were given the option to do so or not, and told there was no implication for their grades as no one would know who did or did not complete the surveys. Consent to participate was obtained by virtue of survey completion after students were provided with information about the class project.

#### Procedures

Students within a single term were approached by a high school classmate and asked to complete a survey for her science research experience project. This occurred in STEM classes where teachers gave permission for the student to distribute her survey. Students were informed that their participation was not mandatory and would not affect their class standing as it was unrelated to any classroom activities. Subjects were asked to complete the survey only one time should they be enrolled in more than one relevant class. The study was described as assessing factors that relate to student learning and performance, particularly in pSTEM fields. Subjects were told to think of the particular class in which the survey was being administered for items that refer to *my class*. Other items referred to their high school in general, by name. There were 36 total items, all rated on 1-6 scales with labels of *Strongly Disagree, Disagree, Somewhat Disagree, Somewhat Agree, Agree, and Strongly Agree*. Items representing the five constructs below were presented in a single random order. Specific items selected were based on past research, with an eye toward minimizing the overall length of the questionnaire.

*pSTEM social belonging* was assessed with five items used in past research (Walton and Cohen, 2007; Lewis et al., 2017; Banchefsky et al., unpublished): *I feel a connection with the community associated with my class; I feel like an outsider in my class* (reverse coded); *I feel like I belong in my class; People in my class accept me; People in my class are a lot like me* (*α* = 0.81).

*pSTEM ability belonging* was measured with a subset of five items from Lewis and Hodges (2015): *I feel similar to the kinds of people who have what it takes to succeed in my class; I’m not certain I “fit in” intellectually in my class* (reverse coded); *When I’m doing work in my class, I feel a sense of competence; I sometimes feel like other students in my class have skills that I don’t* (reverse coded); *I worry that no matter how hard I try, I won’t be able to perform successfully in my class* (reverse coded). Although the full scale had high reliability in samples of college students (Lewis and Hodges, 2015), the five items administered here had poor reliability in this sample (*α* = 0.42). Alpha was improved to 0.61 by omitting *I sometimes feel like other students in my class have skills that I don’t.* Analyses are based on the average of the four items with higher reliability.

*School belonging* was measured with nine items. Five were analogs of social belonging at the class level items, substituting the name of the specific high school in place of *my class* (e.g., *I feel a connection with the community associated with* [name of school]). The remaining three items were *I can relate to my peers at [name of school]; I have considered transferring out of* [name of school] (reverse coded); [name of school] *is the wrong school for me, intellectually* (reverse coded) (*α* = 0.90).

Following Bandura’s (2006) recommendation, *pSTEM self-efficacy* was measured with seven items assessing students’ beliefs about specific capabilities associated with their specific class (Lewis et al., 2017; Banchefsky et al., unpublished): *I am confident that I can demonstrate what I know on exams in my class, I am confident that I can complete homework assignments by myself in my class, I am unable to demonstrate what I learn in my class on exams* (reverse coded), *I am confident that I can perform well on exams in my class, I am not confident that I can learn and understand the concepts taught in my class* (reverse coded), *I am confident that I can complete the class with a B or better*, and *I am confident that I can learn the basic concepts associated with the class* (*α* = 0.86).

*Intentions to Persist in pSTEM* items asked about the likelihood of persisting in the current class, pursuing the topic further in high school and college, and pursuing a career related to the topic. Although the full set of items had good reliability (*α* = 0.91) we became concerned that whereas intentions to pursue the topic in college and beyond are more reflective of personal preference, decisions about high school classes are more constrained (e.g., by state graduation requirements). These latter items are less well-suited to assessing intentions under higher levels of choice so analyses use only the following six items: *I’m interested in knowing more about the subject being taught in this course; I could see myself going into a career related to the subject of this course*; *In college, I plan to major in a field or subject related to this class; After fulfilling my high school requirements, I will not take another course like this* (reverse coded); *I will look into joining/have joined extracurricular activities related to the subject in this class; I would like to pursue subjects similar to the one taught in this course in college* (*α* = 0.91). The additional three items not included in the analyses were: *I look forward to taking more high school courses in this subject; I have considered dropping this course* (reverse coded); *I will not pursue studying this subject after this course is complete* (reverse coded).

For all measures, mean scores were computed, with higher values indicating greater social belonging in pSTEM, ability belonging in pSTEM, school belonging, pSTEM self-efficacy, and intentions to persist in pSTEM.

### Study 1 Results

#### Gender Differences in Belonging, Efficacy, and Intentions

We first assessed the effect of gender on all variables. As shown in **Table 1**, consistent with past research, lower pSTEM social belonging, pSTEM ability belonging, pSTEM self-efficacy, and intentions to persist in pSTEM were reported from females than males. By contrast, general school belonging was equally high among females and males.

**Table 1:** Gender differences in belonging, efficacy, and intentions to persist in pSTEM.

	Social belonging	Ability belonging	School belonging	Self-efficacy	Intentions to persist
Females	4.12 (0.83)	3.86 (0.71)	4.59 (0.89)	4.48 (0.94)	3.21 (1.21)
Males	4.44 (0.74)	4.24 (0.58)	4.70 (0.71)	4.99 (0.76)	4.06 (1.17)
*t* (125)	**2.21**	**3.23**	0.74	**3.23**	**3.96**
*p*	0.029	0.002	0.460	0.002	<0.001


*Mean (and standard deviations in parentheses) for females and males are shown in the top two rows, respectively. The significant values are indicated in bold.*

Although cell sizes are small and the gender distribution is not always equal, we conducted exploratory analyses to see if gender differences in belonging, efficacy, and intentions were moderated by field. We first created three categories of classes: physical sciences (advanced chemistry and Advanced Placement physics; 19 females, 18 males), math (geometry, advanced pre-calculus, Advanced Placement calculus; 26 females, 25 males), and technology (design technology, robotics, Advanced Placement Java; 8 females, 31 males). Separate 2 (gender) × 3 (pSTEM field) between-subjects ANOVAs on each measure revealed only the main effects of gender that replicate results in **Table 1**. There were no main effects or interactions with pSTEM field, indicating the gender differences in **Table 1** occurred to the same degree across these three categories of classes.

#### Predictors of pSTEM Intentions

We next evaluated what predicts intentions to persist in pSTEM separately for females and males. Before doing so, we computed bivariate correlations among all variables, which are shown in **Table 2** (female subjects) and **Table 3** (male subjects). Looking first at what correlates with intentions, for females only pSTEM social belonging was correlated with intentions whereas no variables correlated with intentions for males. The predictors themselves tended to correlate. For females, all predictors except school belonging and self-efficacy were positively correlated, indicating covariation among pSTEM social belonging, pSTEM ability belonging, school belonging, and pSTEM efficacy. For males, all bivariate correlations among the predictors were significant except school belonging with pSTEM efficacy and pSTEM ability belonging. This may indicate more domain-specificity for males, with school belonging not relating to beliefs about specific courses.

**Table 2:** Intercorrelations among belonging, efficacy, and intentions to persist in pSTEM for female subjects.

	Ability belonging	School belonging	Self-efficacy	Intentions to persist
Social belonging	0.347^∗^	0.699^∗∗^	0.376^∗∗^	0.390^∗∗^
Ability belonging		0.292^∗^	0.663^∗∗^	0.129
School belonging			0.225	0.141
Self-efficacy				0.230


*^∗^p < 0.05, ^∗∗^p < 0.01.*

**Table 3:** Intercorrelations among belonging, efficacy, and intentions to persist in pSTEM for male subjects.

	Ability belonging	School belonging	Self-efficacy	Intentions to persist
Social belonging	0.246^∗^	0.558^∗∗^	0.467^∗∗^	0.136
Ability belonging		-0.004	0.537^∗∗^	0.000
School belonging			0.212	-0.091
Self-efficacy				0.148


*^∗^p < 0.05, ^∗∗^p < 0.01.*

We next used regression analyses to evaluate predictors of pSTEM intentions. We initially omitted ability belonging due to its low reliability. Thus, we regressed pSTEM intentions on pSTEM social belonging, pSTEM ability belonging, school belonging, and pSTEM self-efficacy, separately by gender. As can be seen in the left side of **Table 4**, pSTEM social belonging was a significant predictor of intentions to persist for females, with high school females who felt a higher sense of belonging in their current high school pSTEM class expressing greater intentions to engage with pSTEM in the future. By contrast, there were no significant predictors of high school males’ pSTEM intentions (right side of **Table 4**).

**Table 4:** Social belonging, school belonging, and self-efficacy predicting intentions to persist in pSTEM.

	Female subjects			Male subjects		
		
	Standardized beta	*t*	*p*	Standardized beta	*t*	*p*
Social belonging	**0.54**	**2.83**	**0.007**	0.23	1.46	0.15
School belonging	-0.25	-1.40	0.17	-0.24	-1.70	0.09
Self-efficacy	0.09	0.62	0.54	0.09	0.71	0.48


*The significant values are indicated in bold.*

Conclusions are the same when ability belonging is added to the model. pSTEM social belonging remains the only significant predictor of pSTEM intentions for females (left side of **Table 5**), and none of the predictors of males’ pSTEM intentions are significant (right side of **Table 5**). However, as can be been seen in both **Tables 4, 5**, school belonging is a marginal predictor of male’s intentions, with a lower sense of overall school belonging associated with greater intentions to pursue pSTEM.

**Table 5:** Social belonging, ability belonging, school belonging, and self-efficacy predicting intentions to persist in pSTEM.

	Female subjects			Male subjects		
		
	Standardized beta	*t*	*p*	Standardized beta	*t*	*p*
Social belonging	**0.54**	**2.80**	**0.007**	0.24	1.55	0.13
Ability belonging	-0.07	-0.42	0.68	-0.15	-1.11	0.27
School belonging	-0.24	-1.32	0.19	-0.26	-1.86	0.07
Self-efficacy	0.13	0.74	0.46	0.17	1.16	0.25


*The significant values are indicated in bold.*

Results in all regression analyses were largely the same when we included in our dependent measure the additional three intention items that refer to choices under higher levels of constraint. Patterns of significance were identical except for the model predicting males’ intentions from pSTEM social belonging, pSTEM ability belonging, school belonging, and pSTEM self-efficacy. Whereas there were no significant predictors using the narrower measure of intentions, self-efficacy significantly predicted the broader measure of intentions, *b* = 0.51, *t* = 2.08, *p* = 0.041.

### Study 1 Discussion

Study 1 highlights the possible influence of a subjective sense of social belonging on pursuing pSTEM among high school females. As predicted, high school females taking a range of pSTEM classes felt less socially accepted than males in their pSTEM classes. They also felt less belonging in terms of pSTEM ability and lower pSTEM self-efficacy. While ability belonging and efficacy have each been associated with academic persistence, when considered together, social belonging was the only significant predictor of females’ intentions to persist in pSTEM in this high school sample. By contrast, none of the variables we assessed were significant predictors of pSTEM intentions for males. This gender difference in correlates of intentions and actual persistence replicates past research at the college level (Lewis et al., 2017; Banchefsky et al., unpublished).

This leaves the question of what is influencing intentions to persist in pSTEM among high school males. In college samples, course performance (exam scores) rather than belonging predicted pSTEM persistence for males (Lewis et al., 2017), suggesting that whereas females are more influenced by subjective sense of fit, males are more influenced by external markers of content mastery. We were unfortunately unable to obtain any course performance data in the present study so we were unable to evaluate whether this is the case at the high school level as well.

Gender differences in belonging were restricted to feelings of fit (both social and academic) within pSTEM in particular. By contrast, females and males felt an equally high level of belonging at the school level, demonstrating the importance of the more specific pSTEM context when considering gender disparities in pSTEM persistence and achievement. As an additional form of specificity, the results further argue against a general gender-role explanation for the link between belonging and intentions for females in this and other research. Although women are socialized to attend more to social relations than men, only social belonging within pSTEM and not belonging at the school level predicted intentions for females in our sample. Thus, there is no evidence that females in general merely show a closer correspondence between any sense of fit and intentions to persist.

## Study 2

Having established that social belonging relates to intentions to persist in pSTEM in much the same way in high school as in college, we next turn to assessing a second factor that has been identified as affecting pSTEM interest – perceptions about pSTEM fields. We specifically focus on beliefs about the degree to which success in pSTEM favors brilliance over hard work. Because stereotypes about females’ intellect place less emphasis on innate brilliance than comparable stereotypes about males (e.g., Bennett, 2000; Rammstedt and Rammsayer, 2002; Furnham et al., 2006; Bian et al., 2017), females may be selectively discouraged from pursuing fields perceived as requiring innate aptitude. This could occur via self-selection, with females being turned off from fields they perceive to be a poor match for their strengths, or via subtle or blatant discouragement from parents, counselors, and/or teachers.

Assumptions about the requirement of innate brilliance within pSTEM fields were first demonstrated by Leslie et al. (2015), who surveyed faculty, postdoctoral fellows, and graduate students from a range of STEM, social sciences, and humanities disciplines about the degree to which being a top scholar in their field requires raw, innate talent. As predicted, fields with fewer women in them were reported to require more brilliance. Note that this line of research has focused on examining gender disparities across a range of disciplines, not just within pSTEM. The researchers thus showed that even outside of pSTEM, fields with higher field-specific ability beliefs (FABs) such as philosophy had fewer women in them. A subsequent study showed that similar FABs are held by the general public, and that perceptions within this general population sample also predict women’s representation within these fields (Meyer et al., 2015). More recently, a direct relation between a field’s assumed reliance on innate brilliance and decreased interest among women has been demonstrated (Bian et al., 2018). This research showed that women were less interested in pursuing a college major whose students were described as “brilliant,” “smart,” “intelligent,” and “talented” than one whose students were described as “dedicated,” “motivated,” “hardworking,” and “passionate.” By contrast, men’s interest was unaffected by the descriptions.

The purpose of Study 2 was to assess whether high school students similarly perceive a field’s reliance on innate brilliance to covary with its gender distribution. If so, we expect high school students to perceive more male-dominated fields to require more brilliance (Leslie et al., 2015; Meyer et al., 2015). Since pSTEM fields tend to have more men than women, this relation would mean that pSTEM fields should be among those perceived as requiring innate brilliance. We further expect these perceptions to explain gender differences in intentions to pursue the fields, with increases in the need for brilliance associated with relatively lower interest among female than male high school students.

### Study 2 Materials and Methods

#### Subjects

Data were collected from 259 students from the same high school as Study 1. Because we were interested in perceptions of many different fields, there was no requirement that they be enrolled in a pSTEM class. Analyses omitted data from 3 participants reporting non-binary gender identification because this small sample unfortunately precluded reliable inferences about such students. Among the remaining 256 subjects used in data analysis, self-reported race was as follows: among females, 71 identified as White, 7 as Black/African American, 6 as Asian/Asian American, 29 as Latina/Hispanic, and 8 as other; among males, 88 identified as White, 4 as Black/African American, 5 as Asian/Asian American, 31 as Latino/Hispanic, and 7 as other. Data were collected in the school year following the year in which Study 1 was completed. Since data were collected anonymously in both studies, we have no way of determining if any subjects participated in both studies. The different research questions assessed in the two studies suggests it would not be problematic if someone participated in both.

#### Procedures

A high school classmate approached students within a single term and asked them to complete a survey for her science research experience project. Those who provided verbal agreement were presented with a survey asking their perceptions of different fields and told that if a subject area was unfamiliar to them, they should answer based on what they do know about the topic. Subjects were first asked to rate the FABs of each of seven disciplines. We identified 21 STEM, social science, and humanities disciplines that we expected high school students to have exposure to and knowledge of from the larger list of disciplines used by Leslie et al. (2015) and Meyer et al. (2015). Following Meyer et al. (2015), rather than having all subjects rate all disciplines, we created three versions of the survey, each with three STEM, two social science, and two humanities fields. Version A (*n* = 114) asked about chemistry, computer science, statistics, political science, psychology, education, and Spanish. Version B (*n* = 75) asked about earth sciences, engineering, biology ^2^, sociology, art history, history, and philosophy. Version C (*n* = 67) asked about physics, mathematics, neuroscience, economics, anthropology, english literature, and music theory and composition.

Field-specific ability beliefs were assessed with the four items from Leslie et al. (2015): *Being a top scholar of this field requires a special aptitude that just can’t be taught; If you want to succeed in this field, hard work alone just won’t cut it; you need to have an innate gift or talent; With the right amount of effort and dedication, anyone can become a top scholar in this field; When it comes to this field, the most important factors for success are motivation and sustained effort; raw ability is secondary* (Version A *α* = 0.90, Version B *α* = 0.88, and Version C *α* = 0.90).

Subjects were then asked their intentions to pursue each discipline. For the sake of brevity, we selected four of the intentions items used in Study 1: *After fulfilling my high school requirements, I will not take another course in this discipline* (reverse coded); *I’m interested in knowing more about this subject; I would like to pursue subjects similar to this in college; I could see myself going into a career related to this subject* (Version A *α* = 0.86, Version B *α* = 0.79, Version C *α* = 0.85). FABs and intentions were rated on the same 1–6 scale used in Study 1. Finally, subjects provided their gender and race and then indicated their exposure to different disciplines by checking from a list of all courses offered at their high school the courses they had taken.

### Study 2 Results

#### Beliefs About Brilliance and Women’s Representation

**FIGURE 1:**
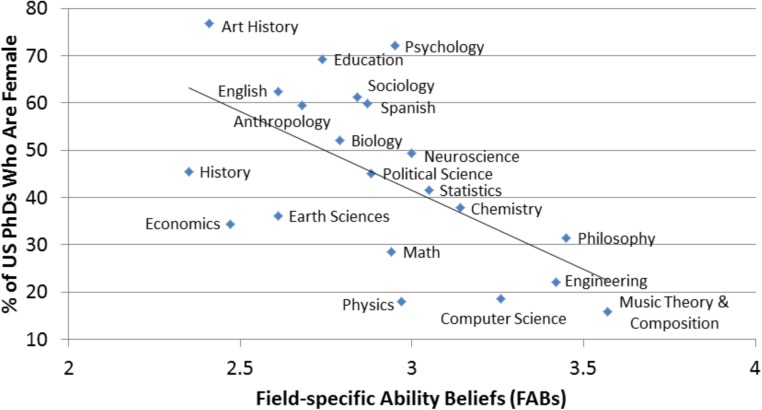
The relationship between mean beliefs about the necessity of brilliance for success in a field and the percent of women in that field, operationalized as percent of women earning Ph.D’s in 2011. Field of study is the unit of analysis.

We assessed our first hypothesis that high school students perceive male-dominated fields to require more brilliance in two ways. The first method directly replicated the correlational analysis of Leslie et al. (2015) and Meyer et al. (2015). We first calculated for each field a mean FAB score across all the subjects who rated that field (and across all four FAB items completed by each subject), with higher scores indicating more assumed brilliance. These means were then correlated with the percent of women in a field, operationalized as percentage of women earning a Ph.D. (National Science Foundation [NSF], 2011) (cf. Leslie et al., 2015). Field was thus the unit of analysis. As can be seen in **Figure 1**, as assumed brilliance increased, the percent of women in the fields decreased, *r*(19) = -0.598, *p* = 0.004.

Our second analysis assessing this same hypothesis used a linear mixed model, a potentially more powerful approach because it explicitly models rather than collapses across subject variance. Both subjects and fields were treated as random factors, and we estimated random slopes and intercepts for each subject as well as random slopes for each field. Another benefit of this analytical approach is that it allows us to explicitly model theoretically relevant subject variables. Meyer et al. (2015) argued that within a general population, those who had attended college show a stronger relationship between FABs and women’s representation because they have greater exposure to the fields in question. To similarly assess the effect of exposure within our sample, we quantified experience with pSTEM classes in particular. This was done by computing the proportion of pSTEM classes among all classes subjects reported taking in high school. All predictors were mean-centered prior to analysis. Models initially included subject gender and its interactions with FABs and proportion of pSTEM classes taken; there were no significant effects of subject gender, indicating that the effects reported below hold for both females and males (all *p*’s > 0.25); for parsimony, the models presented here omit all subject gender terms.

**FIGURE 2:**
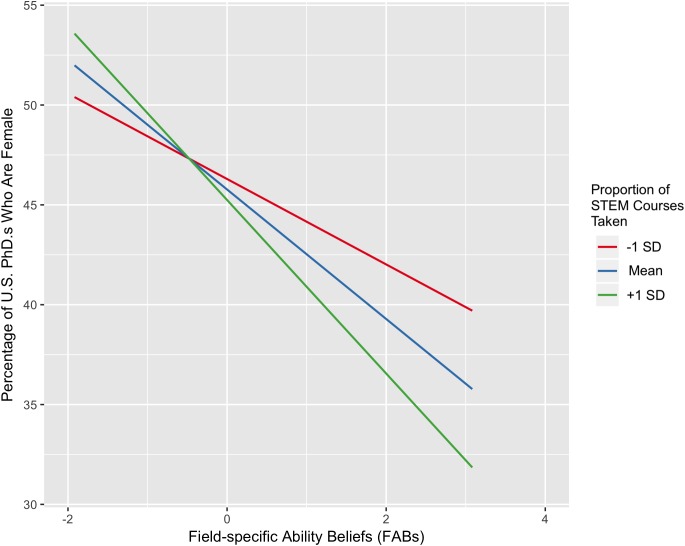
The relationship between beliefs about the necessity of brilliance for success in a field and the percent of women in that field, operationalized as percent of women earning Ph.D’s in 2011. Both subjects and field are random effects. The *green* and *red lines* represent the predicted slope for students 1 standard deviation above and below the mean in proportion of pSTEM courses taken, respectively; the *blue line* depicts the predicted slope for students at the mean of proportion of pSTEM courses taken. FABs is mean-centered.

As predicted, the more strongly students endorsed the necessity of innate brilliance for success in a given field, the smaller the percent of women in that field (*B* = -0.17, *p* = 0.017). Also as predicted, this effect was qualified by a significant interaction with pSTEM class exposure such that the negative relationship between FABs and percent women in the field was stronger among students who had taken a greater proportion of pSTEM classes (*B* = -0.05, *p* = 0.027, see **Figure 2**).

#### Beliefs About Brilliance and Gender Differences in Intentions to Pursue the Field

We next assessed whether perceiving the need for brilliance differentially discourages females from pursuing the field. To do this, we computed the average reported intentions of pursuing each field in the survey for female and male students, then calculated an intention gender difference score for each field by subtracting the average intention to pursue that field among male students in the sample from the average intention among female students in the sample. Higher scores therefore indicate higher intentions among the females in our sample. We then used FAB scores to predict the intention gender difference scores in a linear mixed effects model, treating subjects and fields as random and estimating random slopes and intercepts for each subject as well as random slopes for each field. As in the prior model, pSTEM class experience was also included and initial models including subject gender and all its interactions revealed no significant effects of gender (all *p*’s > 0.37), so the results reported here omit subject gender effects.

**FIGURE 3:**
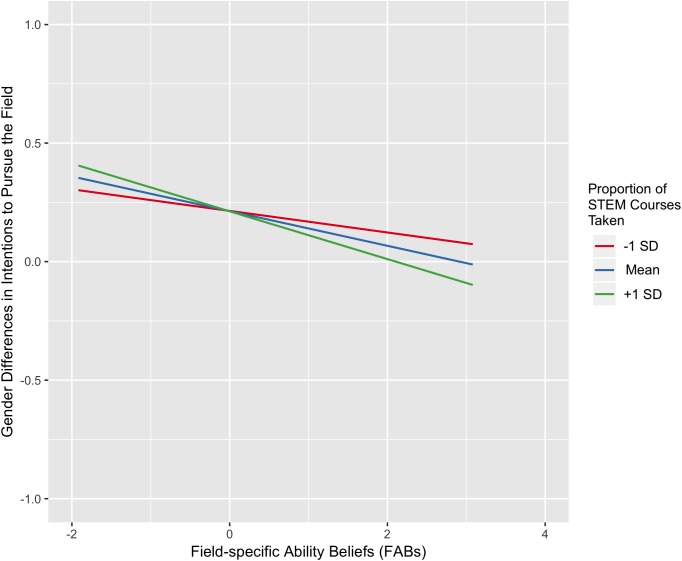
The relationship between beliefs about the necessity of brilliance for success in a field and the difference between mean female and male students’ intent to pursue that field. Higher values on intentions indicates higher intentions among female as compared to male subjects. The *green* and *red lines* represent the predicted slope for students 1 standard deviation above and below the mean in proportion of pSTEM courses taken, respectively; the *blue line* depicts the predicted slope for students at the mean of proportion of pSTEM courses taken. FABs is mean-centered.

The more strongly a field was believed to require innate aptitude, the more negative the female–male intention difference; that is, at higher levels of perceived innate aptitude, the difference shifted toward greater intention to pursue a field among males relative to females (*B* = -0.16, *p* = 0.029). There was also a significant interaction with pSTEM class experiences, such that this effect was stronger among students who had taken a greater proportion of pSTEM classes (*B* = -0.05, *p* = 0.022; see **Figure 3**).

### Study 2 Discussion

High school students are sensitive to the association between a field’s gender distribution and beliefs about the degree to which brilliance is required for success. They not only perceive fields with more men in them to require more brilliance, but gender disparities in their interest in these fields are associated with assumptions about brilliance; those fields assumed to require more brilliance are the ones in which males express greater intentions to pursue them relative to females. Moreover, having more experience with pSTEM fields, many of which have very low representations of women and high expectations of brilliance, increases both relationships. These results extend prior research in showing that the “male = brilliance” assumption is present not only among practitioners in these fields (Leslie et al., 2015) or adults in the general population (Meyer et al., 2015), but also high school students facing an important structural transition that will influence their career trajectory. It is also interesting to see that within the relatively constrained window of pSTEM classes in high school, greater experience with pSTEM is associated with stronger effects deriving from the “male = brilliance” assumption. It seems possible this relation could arise from students acquiring this association through their experiences in pSTEM classes, but it is also possible students predisposed to this orientation are attracted to pSTEM classes.

In addition to replicating the relation between FABs and women’s representation, there was also similarity in the relative positioning of many fields compared to the adult samples in Leslie et al. (2015) and Meyer et al. (2015). For example, both the high school students and adults rated art history and history among the lowest in FABs, but engineering and music theory and composition among the highest (see **Figure 1**). With caveats about comparing across samples in mind, we note differences in the positioning of mean FABs for math. In our sample, the high school students rated math as middling in its requirement of brilliance, but among both practitioners and the general adult population, math was rated as among the highest in FABs. To the degree that FABs affects students’ interest in these fields, it would be interesting to investigate if this represents a general change in how the field is being portrayed to students, something about the experiences of our particular sample (e.g., particular teachers they are exposed to), and/or the exemplars that are brought to mind (e.g., their conception of math may be tied to particulars of their course requirements). There are also teacher training implications if perceptions of brilliance can be influenced by how the subject matter is presented (e.g., see discussions of the benefits of encouraging a malleable view of ability (e.g., Dweck, 2008)). If this is a more general shift among students in this age range, it would also be interesting to track whether this facilitates students’ interest in pursuing math beyond high school.

## General Discussion

Research at higher levels of education shows that both subjective self-perceptions and perceptions about pSTEM fields can influence students’ interest in pSTEM. Although the educational context in high school differs from college in many ways, our data suggest that both processes could selectively dissuade high school females from pursuing pSTEM career paths relative to males. Study 1 showed that high school females taking a wide range of pSTEM classes on average feel less acceptance and fit within their pSTEM classes relative to their male peers, and for females only, pSTEM social belonging is positively associated with intentions to pursue pSTEM. Study 2 showed that high school students perceive many pSTEM fields as requiring innate brilliance, and assumptions about the need for brilliance within a field are associated with relatively lower intentions to pursue those fields among female than male high school students. As the students in our sample would soon be either transitioning to the work force or selecting majors to pursue in higher education, their responses provide a window into how the cumulative effects of individual experiences and the broader cultural context could shape interest in pSTEM fields.

We separately assessed subjective self-perceptions and perceptions about the field in different studies but expect they are ultimately related. Linking a field with stereotypically masculine characteristics such as brilliance may be one of many factors that contribute to lower belonging among females (cf. Cheryan et al., 2017). Along with extant research and theorizing, these results point to the importance of interrogating the full context of pSTEM education – who teaches it, how it is taught, what messages are subtly and no-so-subtly being conveyed – in order to understand gender disparities. Moreover, the similarity of our results to those obtained with college students suggests strategies and interventions effective for boosting pSTEM interest among females at higher levels of education may also be effective at the high school level if appropriately integrated into the context (e.g., exposure to female role models; increases in belonging; Murphy et al., 2007; Stout et al., 2011).

### Limitations

The correlational design of both studies is an important limitation, precluding conclusions about the causal relations among the variables measured. Extant theory and research suggest likely causal relations – for example, a longitudinal study shows that belonging measured at the beginning of a course predicts actual pSTEM persistence in subsequent terms (Lewis et al., 2017) and experimental research shows that describing students in a college major in terms of brilliance rather than their propensity to work hard selectively decreases women’s interest in those majors (Bian et al., 2018) – but we cannot conclude that belonging and FABs measured here will necessarily affect pSTEM persistence among these students. A second limitation is that data were only obtained from a single school; generalizability has yet to be established. Finally, limitations in survey length prevented us from assessing additional factors previously demonstrated to explain gender disparities in pSTEM pursuit. Perhaps not surprisingly, there are a number of other factors associated with pSTEM performance and/or persistence, including outcome expectations (e.g., Lent et al., 1996), prior pSTEM course experience (e.g., Chachashvili-Bolotin et al., 2016), self-perceived ability (Correll, 2001; Ma, 2011), peer influences (e.g., Crosnoe et al., 2008), role models (Dasgupta, 2011), and beliefs about the malleability of ability (Blackwell et al., 2007; Dweck, 2008). The relevance of other, unmeasured variables is particularly highlighted for male subjects in Study 1. None of the four variables we assessed significantly predicted pSTEM intentions, suggesting other, unmeasured variables account for pSTEM persistence among males. We nevertheless think the studies presented here have value for showing that subjective sense of belonging and perceptions about pSTEM fields *can* influence high students’ interest in pSTEM (with social belonging being particularly relevant for females), highlighting processes to be considered within the high school pSTEM educational context.

### Implications

The present results suggest that reducing social marginalization and changing perceptions about pSTEM careers may be ways to increase the representation of girls and women in pSTEM. With respect to social marginalization, extant research unfortunately suggests that many factors contribute to women’s relatively lower levels of belonging in pSTEM, including negative cultural stereotypes about women’s abilities and the dearth of female role models within pSTEM (e.g., Dasgupta, 2011; Cheryan et al., 2017). These are each difficult to change, potentially implying a poor prognosis for improving females’ social belonging. Fortunately, other research shows that changing underlying causes is not the only route for increasing belonging among marginalized groups. Walton et al. (2015), for example, show that a brief social belonging intervention designed to provide students with a nonthreatening narrative framework for interpreting adversity benefits female engineering majors, specifically increasing their engineering GPA, friendships with male engineers, and perceptions about the manageability of adversity (see also Yeager et al., 2016; but for null results, see Broda et al., 2018).

Less is known about the causes of the FABs assessed in Study 2. Practitioners within the fields are suggested as one potential source (Leslie et al., 2015); congruent with this, the negative association between FABs and women’s representation in a field is stronger among adults with college exposure to those fields – and therefore, exposure to its practitioners – compared to those with no college exposure (Meyer et al., 2015). Study 2 similarly found that FABs were more strongly correlated with the gender representation within a field and gender differences in intent to pursue a field among students who had taken more pSTEM classes. The origin of the brilliance beliefs among a field’s practitioners, though, is still not clear. However, like belonging, interventions are possible even if the underlying causes are not fully understood and/or might be difficult to change. The large field studying mindsets points to the benefits of encouraging the view that ability is an attribute that can be developed rather than a fixed trait (Blackwell et al., 2007; Walton et al., 2015; Yeager et al., 2016; Broda et al., 2018). These interventions currently focus on improving students’ individual outcomes via beliefs about their own ability, but the current wide-scale emphasis on a malleable mindset within American primary and secondary education could plausibly color students’ views about others as well. This could potentially include beliefs about entire fields of study by promoting a general view that achievement within any domain is possible through hard work. We noted earlier that our subjects seem to perceive math as requiring less innate brilliance than prior samples of adults. It is possible that being educated within a culture that currently emphasizes the malleability of ability may contribute to this, something which may warrant additional empirical attention.

## Data Availability

The raw data supporting the conclusions of this manuscript will be made available by the authors, without undue reservation, to any qualified researcher.

## Ethics Statement

Both studies report analyses of existing data collected anonymously by a high school student for educational purposes as part of a requirement for a research experience class; such class projects are not considered research by the University of Colorado IRB. After completion of the class projects, the first author and the student who completed the project discussed disseminating the findings, at which point the IRB at the University of Colorado was consulted again. They determined that analyses of these existing anonymous data do not constitute research involving human subjects as defined by the US Department of Health and Human Services and Federal Drug Administration.

Written informed consent was not obtained since both studies were conducted originally for educational rather than research purposes. At the time surveys were completed, students were simply asked if they wanted to complete an anonymous survey for a fellow student’s research experience class. They were given the option to do so or not, and told there was no implication for their grades as no one would know who did or did not complete the surveys. Consent to participate was obtained by virtue of survey completion after students were provided with sufficient information about the class project.

## Author Contributions

TI conceived and planned the studies in consultation with Sydney Fisher as part of a research experience class project. TI and EM analyzed the data and wrote the manuscript.

## Conflict of Interest Statement

The authors declare that the research was conducted in the absence of any commercial or financial relationships that could be construed as a potential conflict of interest.

## Footnotes

1 For clarity, the term STEM will be used when discussing past research that did not differentiate among different science fields, and pSTEM will be used only for studies with a specific focus on physical sciences within the category of science.

2 Leslie et al. (2015) and Meyer et al. (2015) listed evolutionary and molecular biology as separate fields. We thought high school students would be more familiar with a general biology category rather than specific sub-disciplines.
